# DTA-SiST: de novo transcriptome assembly by using simplified suffix trees

**DOI:** 10.1186/s12859-019-3272-9

**Published:** 2019-12-24

**Authors:** Jin Zhao, Haodi Feng, Daming Zhu, Chi Zhang, Ying Xu

**Affiliations:** 1 School of Computer Science and Technology, Shandong University, Binhai Road, Qingdao, Shandong People’s Republic of China; 2 Department of Medical and Molecular Genetics and Center for Computational Biology and Bioinformatics, Indiana University, Indianapolis, IN USA; 3 Department of Biochemistry and Molecular Biology, University of Georgia, Athens, GA USA

**Keywords:** Alternative splicing, RNA-seq, De novo assembly

## Abstract

**Background:**

Alternative splicing allows the pre-mRNAs of a gene to be spliced into various mRNAs, which greatly increases the diversity of proteins. High-throughput sequencing of mRNAs has revolutionized our ability for transcripts reconstruction. However, the massive size of short reads makes de novo transcripts assembly an algorithmic challenge.

**Results:**

We develop a novel radical framework, called DTA-SiST, for de novo transcriptome assembly based on suffix trees. DTA-SiST first extends contigs by reads that have the longest overlaps with the contigs’ terminuses. These reads can be found in linear time of the lengths of the reads through a well-designed suffix tree structure. Then, DTA-SiST constructs splicing graphs based on contigs for each gene locus. Finally, DTA-SiST proposes two strategies to extract transcript-representing paths: a depth-first enumeration strategy and a hybrid strategy based on length and coverage. We implemented the above two strategies and compared them with the state-of-the-art de novo assemblers on both simulated and real datasets. Experimental results showed that the depth-first enumeration strategy performs always better with recall and also better with precision for smaller datasets while the hybrid strategy leads with precision for big datasets.

**Conclusions:**

DTA-SiST performs more competitive than the other compared de novo assemblers especially with precision measure, due to the read-based contig extension strategy and the elegant transcripts extraction rules.

## Background

Alternative splicing plays an important role in regulating gene expression and producing diversity of proteins. Through alternative splicing, the pre-mRNAs of a gene can be spliced into various mRNAs, which results in the large difference between the number of genes and that of proteins [1, 2]. A transcript is defined as a mature mRNA that encodes protein. We call the set of all the transcripts the transcriptome. The transcriptome can be seen as a precursor of the proteome, i.e., the entire set of proteins expressed by a genome. Transcriptome reconstruction is an important mean for studying cell phenotype and function [3]. However, this task is quite nontrivial. For the time being, we only know a small part of the landscape of alternative splicing of some species.

The high-throughput sequencing has revolutionized our ability to study many challenging issues such as motif finding, DNA/RNA-protein interaction, ribosome profiling, small RNA expression profiling, transcripts assembly, and disease diagnosis [4–9]. The RNA sequencing (RNA-seq) offers a great opportunity to identify the expressed transcripts.

There are generally two alternative computational strategies for transcriptome assembly problems: genome-guided approaches such as Scallop [10], Cufflinks [11], StringTie [12], CIDANE [13], Scripture [14], IsoLasso [15], and TransComb [16], and de novo transcripts assembly approaches such as Trinity [17], Oases [18], SOAPdenovo-Trans [19], IDBA-Tran [20], BinPacker [21], Bridger [22], ABySS [23], and IsoTree [24]. Genome-guided assemblers are generally more accurate than de novo assemblers when the high-quality reference genome is available. However, the high-quality reference genome is not always available. In this situation, de novo transcriptome assembly is required. In this paper, we mainly consider de novo transcriptome assembly methods.

Existing strategies for transcriptome assembly usually adopt the following scheme: first constructing graphs based on the RNA-seq reads, and then extracting paths from the graphs to represent plausible transcripts. Various algorithms are employed to recover transcript-representing paths. For example, Cufflinks [11] and Bridger [22] employ the minimum path cover algorithm to extract the minimum number of paths that cover all the vertices. StringTie [12] applies the network flow algorithm on the splicing graphs to recover all the possible transcripts. Trinity [17] extracts the sufficiently covered paths from the compact graphs based on a brute-force enumeration strategy. BinPacker [21] models the paths in the splicing graphs as a set of trajectories of items by solving a series of bin-packing problems. Note that Cufflinks and StringTie belong to genome-guided approaches. They usually start by aligning reads to a reference genome, and then construct graphs according to alignment results. Trinity, Bridger, and BinPacker are de novo transcriptome assembly approaches, and they build graphs solely based on the overlaps of *k*-mers (*k*-character substrings of read sequences). To investigate the influence of path extracting strategies on the assemblers’ performances, DTA-SiST developed two strategies: a depth-first enumeration strategy and a hybrid strategy based on length and coverage. The first strategy aims to distinguish as many as possible transcripts while the second tries to target candidates more accurately. DTA-SiST implemented these two strategies and compared them with the state-of-the-art assemblers.

Historically, the de novo assembly approaches mostly rely on the pioneering works on de Bruijn graphs [25], including Trinity [17], SOAPdenovo-Trans [19], Oases [18], IDBA-Tran [20], and Trans-AByss [23]. Recently, Bridger [22], BinPacker [21], and IsoTree [24] applied splicing graphs [26] to represent alternative splicing. Both de Bruijn graphs and splicing graphs are usually constructed by extending contigs with *k*-mers. Each node in a de Bruijn graph represents a *k*-mer while a node in a splicing graph usualls to an exon. Hence, the number of nodes in a splicing graph is far less than that of the nodes in a de Bruijn graph, which makes the models based on splicing graphs more tractable.

The *k*-mer-based extension strategies extend a contig through a *k*-mer whose first (or last) *k*−1-character substring is exactly the same as the last (or first) *k*−1-character substring of the contig. For convenience, we say that the *k*-mer has a *k*−1-character *overlap with the contig’s ternimus*. For example, suppose the contig sequence is ACATCG, and the *k*-mer set contains the *k*-mers of TACA, ACAT, CATC, ATCG, and TCGG. Since the last 3-character substring of TACA is exactly the same as the first 3-character substring of the contig, i.e., the *k*-mer of TACA has a 3-character overlap with the contig’s terminus, the contig can be extended by TACA to TACATCG. Similarly, the first 3-character substring of TCGG is exactly the same as the last 3-character substring of the contig. Through TCGG, the contig of TACATCG can be extended to TACATCGG. By applying a hash table to hold the *k*-mers with their original reads’ IDs as their corresponding values, the *k*-mer-based strategies can find the candidate *k*-mers quickly. However, since a *k*-mer may originate from quite a few different reads which may easily lead to a wrong extension, these strategies deny making full use of the information of the whole nucleotides arrangement in each read. Although the *k*-mer-based contig extension strategy has been widely used in de novo assemblers, both the accuracy and sensitivity are still far from meeting the requirement.

Some multiple-*k* strategies have been developed such as Oases-M [18], IDBA-Tran [20], and Bridger-M [22], but the problem is not solved basically since these works just intuitively tried several *k* values one after another without fully considering the variances of the lengths of the actual overlaps between reads. In our last work IsoTree [24, 27], we proposed a method to find the candidate reads that have the longest overlap to the contig’s terminus. But the method needs to try all the candidate reads that may have overlaps of lengths between *L*−1 and *x* with the contig’s terminus, which is time consuming and a little tedious. Here, *L* is the length of reads, and *x* is the length of the longest overlap between the contig’s terminus and the available reads. In this work, we introduce a more straightforward contig extension strategy that extends a contig by the read that has the longest overlap with the contig’s terminus. Through the suffix trees of reads, we can find the candidate reads that have the longest overlap with a contig’s terminus in *O*(*L*) time by just scanning the first (for left extension) or the last (for right extension) *L*−1 characters of the contig once.

In the rest of this work, the methods of constructing suffix trees and splicing graphs as well as the algorithms of extracting transcript-representing paths from splicing graphs are first introduced. Then, the experimental results and discussions are presented. The final conclusions are given in the end.

## Methods

### Suffix tree construction

Traditional *k*-mer-based extension strategies build overlapping *k*-mers from reads and extend contigs by *k*-mers, which can only guarantee *k*−1-character overlaps between contigs and *k*-mers. The whole nucleotides arrangements of reads are usually ignored in *k*-mer-based extension strategies. Obviously, extending contigs by reads is more credible than extending contigs by *k*-mers, especially for long reads. However, it is much more complicated to find a read that holds the longest overlap to the current contig’s terminus than to find a *k*-mer that overlaps the current contig’s terminus by exactly *k*−1 characters. The latter can be conveniently realized by using hash tables. Some multiple-*k* strategies have been developed to overcome the shortage of single-*k* strategies by trying several *k* values. But these intuitive designs were far from the real sense that reads may overlap with each other by different numbers of characters. In order to make full use of nucleotide arrangements of reads, DTA-SiST presents a read-based contig extension strategy. The main point is that DTA-SiST applies a suffix tree structure to quickly find the candidate reads.

**Fig. 1:**
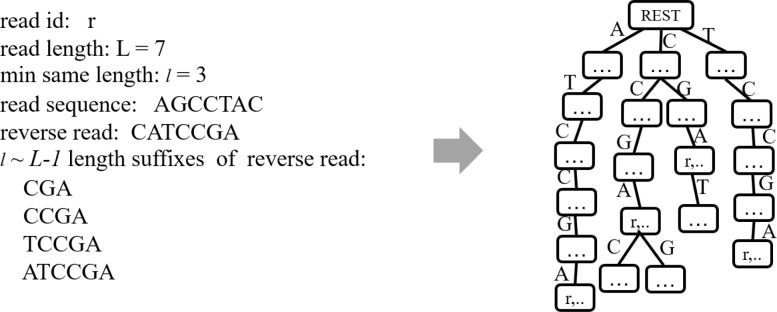
Right extension suffix tree. An example for adding the suffixes of a reverse read to the right extension suffix tree

To facilitate the contig extension from 5’ to 3’, DTA-SiST builds a suffix tree, called right extension suffix tree (REST). Although the beginning of the maximum overlap between the current contig’s 3’ terminus and the candidate reads is unknown, the ending of the maximum overlap is known. The maximum overlap is actually a prefix of the candidate reads. Consequently, DTA-SiST reverses all the read sequences and constructs a suffix tree for the *l*∼*L*−1 character suffixes of all the reverse reads, where *l* denotes the predefined minimum overlap length and *L* represents the read length. In the right extension suffix tree, the path from the root node to each node represents a substring of some reverse reads. If the *x*-length (*l*≤*x*≤*L*−1) path from the root node to the node *v* represents a suffix of the reverse read of *r*, DTA-SiST stores the read id *r* in the node *v* (as shown in Fig. 1).

**Fig. 2:**
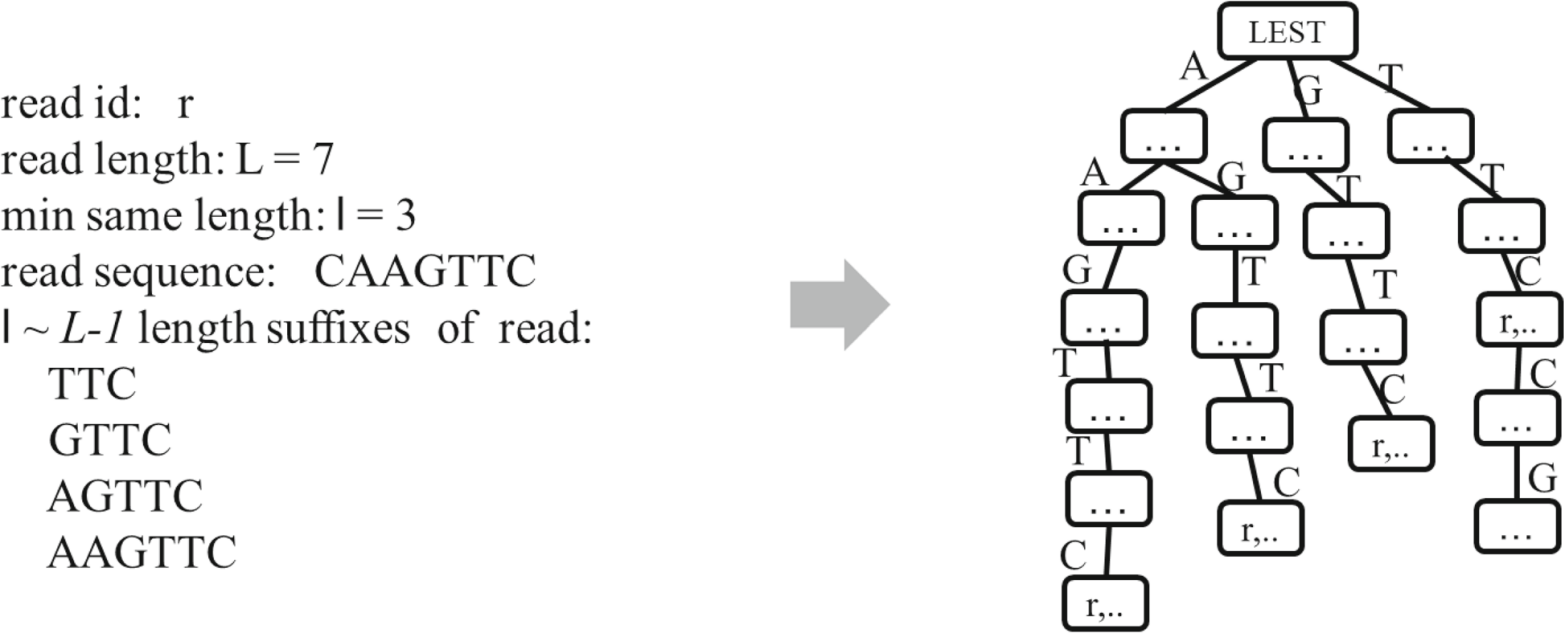
Left extension suffix tree. An example for adding the suffixes of a read to the left extension suffix tree

Similarly, DTA-SiST constructs a left extension suffix tree (LEST) to facilitate the contig extension from 3’ to 5’. In this case, the maximum overlap is actually a suffix of candidate reads and the start point of the overlap is known. Hence, the left extension suffix tree consists exactly of the *l*∼*L*−1 character suffixes of all the reads. An example for adding a read to the left extension suffix tree is shown in Fig. 2.

Through the suffix trees constructed with the above method, DTA-SiST gets the candidate reads that hold the longest overlap with current contig’s terminus in *O*(*L*) time by scanning prefix of the contig (for left extension, right extension is processed similarly) along the edges in the left extension tree as far as possible and then moving back to find the reads’ IDs stored in the nearest node. Suppose there are totally *N* reads. In theory, the suffix trees will take up *O*(*M*+*N*(*L*−*l*)) space, where *M* is the size of all the suffixes. Notice that *M* can be up to *O*(*N*(*L*+*l*)(*L*−*l*)) supposing that all the suffixes are different. The space of the suffix trees can be divided into two parts, one is to store the reads’ IDs, and the other is to store the tree structure. In order to reduce the space consumed in storing reads’ IDs, we compress the reads with the same sequence into one read and assign them the same ID. After numerous experiments, we found that many vertices and reads’ IDs stored in the suffix trees are useless. The reason is that not all the suffixes (or prefixes) of reads can match contigs’ 5’ (or 3’) terminuses, and thus they will never be used in the left (or right) extension. If the suffix (or prefix) of a read can be used in the left (or right) extension, this suffix (or prefix) must be a prefix (or suffix) of another read. Based on this observation, we construct the simplified suffix trees with the following steps. The idea is that we first extract all the *L*−1-character prefixes of all the reads. Then we check and store the suffixes with lengths of *l,l*+1,...,*L*−1 that overlap with these prefixes and thus get the left extension suffix tree LEST. Finally, we check and store the prefixes with lengths of *l,l*+1,...,*L*−1 that overlap with the suffixes in the LEST and thus get the right extension suffix tree REST. As defined before, the prefixes stored in the REST are actually their reversals.

**step 1:** Initialize the left extension suffix tree LEST structure by the *L*−1-character prefixes of all the reads. If a suffix is usable in the left extension, it must overlap with a *L*−1-character prefix of reads. Hence, the tree constructed with the *L*−1-character prefixes covers all the usable suffixes.

**Fig. 3:**
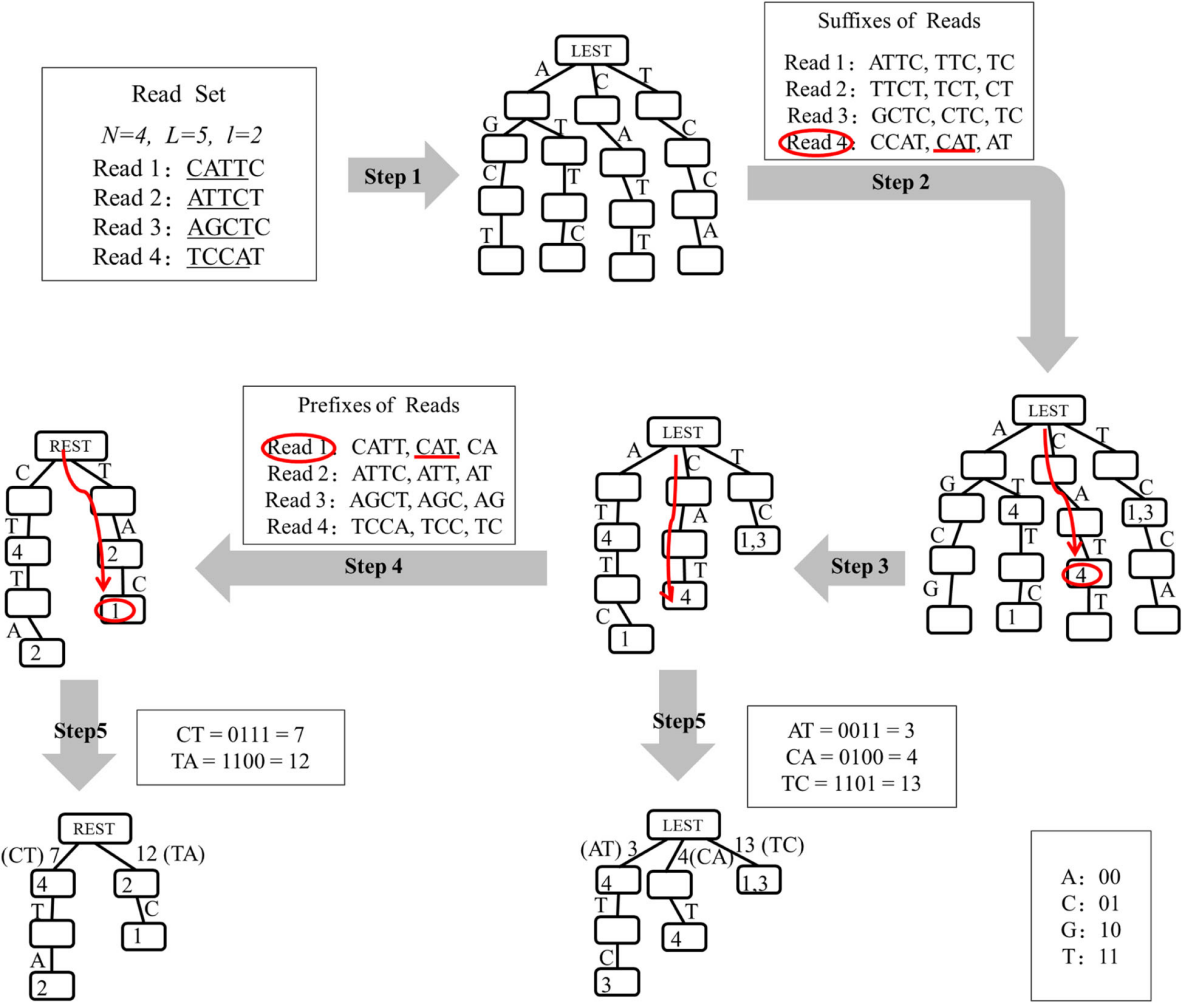
Simplified suffix tree. An example for constructing the simplified suffix trees. In this example, there are total of four reads. The read length is 5*bp*, and the minimum overlap length is 2*bp*

Figure 3 gives an example to construct the simplified suffix trees. As shown in this figure, there are total of four reads, i.e., Read1 as sequence CATTC, Read2 as sequence ATTCT, Read3 as sequence AGCTC, and Read4 as sequence TCCAT. The length of these reads is 5*bp* (*L*=5), and the minimum overlap length is 2*bp* (*l*=2). The 4-character prefixes of these reads are used to construct the tree structure.

**step 2:** Add reads’ IDs to the left extension suffix tree. If one suffix (whose length is between *l* and *L*−1) of read *r* follows a path from the root node to node *v* in the LEST, DTA-SiST stores the read id *r* in the node *v*.

In the above example (Fig. 3), the suffixes with lengths 4,3,2 of Read1 are ATTC, TTC, and TC, respectively. DTA-SiST first scans the 4-character suffix (ATTC) in the left extension suffix tree. DTA-SiST finds that there is a path from the root node in the tree that exactly corresponds to ATTC, and DTA-SiST adds the ID of Read1 into the left extension suffix tree. Then, DTA-SiST scans the 3-character suffix (TTC) and the 2-character suffix (TC) in the left extension suffix tree successively. Since there is no path from the root node in the left extension suffix tree marked as TTC while there is one marked as TC, DTA-SiST adds the ID of Read1 only in the node corresponding to path TC. Similarly, DTA-SiST adds the IDs of the other reads into the left extension suffix tree.

**step 3:** Trim the left extension suffix tree. DTA-SiST iteratively deletes the leaf nodes without reads’ IDs.

As shown in Fig. 3, the leaf nodes without reads’ IDs are iteratively removed. Through the trimming operations, the number of nodes in the left extension suffix tree is reduced from 16 to 10.

**step 4:** Construct the right extension suffix tree REST based on the left extension suffix tree. DTA-SiST scans each *x*-character (*l*≤*x*≤*L*−1) prefix of all the reads to check whether it matches a path from the root to a node with some stored read IDs in the left extension suffix tree. If yes, DTA-SiST will reverse the prefix, add the reversal sequence into the right extension suffix tree, and store the corresponding read’s ID in the corresponding node.

In the above example (Fig. 3), the 2,3,4-character prefixes of Read1 are CA, CAT, and CATT, respectively. DTA-SiST first scans the 2-character prefix (CA) in the left extension suffix tree. Although there is a path from the root node marked CA, but the ending node of the path contains no IDs which means that CA is only part of a usable suffix. In this case, DTA-SiST will not add the ID of Read1 but continue to scan the 3-character prefix (CAT). Since CAT matches a path from the root node in the left extension suffix tree and the ending node of the path contains reads’ IDs, DTA-SiST reverses CAT to TAC and adds TAC as well as the ID of Read1 to the right extension suffix tree. Then, DTA-SiST scans the 4-character prefix (CATT) in the left extension suffix tree similarly. The prefixes of the other reads are processed in the same way.

**step 5:** Compress the left extension suffix tree and the right extension suffix tree. In order to save the space and improve the speed, each *l*-character path from the root node is compressed into one node and stored as a 64-bit unsigned integer each.

As shown in Fig. 3, through the compressing operation, the number of nodes in the left extension suffix tree is reduced from 10 to 7.

With these improvements on the suffix trees, the memory cost by DTA-SiST is comparable to that cost by the hash table data structure as shown in Table 1. Note that in theory, the hash table for single *k*-mer representation takes up a memory of *O*(*N*(*L*−*k*+1)), and the simplified suffix tree takes up a memory of *O*(*N*^′^(*L*−*l*)), where *N*^′^(*N*^′^≤*N*) is the number of reads with usable suffixes or prefixes. As mentioned above, the memory of the trees is spent in storing the tree structures and reads’ IDs. Since the initial left extension suffix tree structure is constructed from the *L*−1-character prefixes of all the reads and that the *l*-character paths from the root node are compressed into one node each, there are at most *O*(*N*^′^(*L*−*l*)) nodes in the tree. The memory that is used to store the reads’ IDs can be up to *O*(*N*^′^(*L*−*l*)) supposing that all the suffixes of these *N*^′^ reads are usable. The same results can be drawn for the right extension suffix tree. Note that the hash table for multiple *k*-mer representation takes up a memory of *O*(*M*^′^+*N*(*L*−*l*)^2^), where *M*^′^ is the size of all the *l*+1,*l*+2,...,*L*-mers if we use hash table of all these substrings to find the *k*-mers that have the *longest* overlaps with the contigs’ terminuses. Notice that *M*^′^ is quite larger than *M*, the size of all the *l,l*+1,...,*L*−1-suffixes. We compared the memories occupied by the suffix trees, the hash table of *k*-mers with single length of *l*+1, and hash table of *k*-mers with multiple lengths of *l*+1,*l*+2,...,*L*.

**Table 1:** Comparison of memory occupied by different strategies

Reads	50bp	75bp	100bp	50bp	50bp
	0.1million	0.1million	0.1million	0.5million	1million
Suffix tree	68.3M	143.5M	220.5M	85.1M	94.3M
Single-*k*	20.8M	34.7M	49.2M	78.5M	149.3M
Multiple-*k*	286.0M	572.9M	899.5M	445.9M	640.6M

Table 1 shows that the simplified suffix tree strategy occupies comparable memory as that used by the single-*k* strategy while the multiple-*k* strategy costs much more. Notice that multiple-*k* strategy is impractical in finding the best candidate that has the longest overlap with the contig’s terminus due to its memory usage for real data (notice that multiple-*k* strategies such as Oases-M [18], IDBA-Tran [20], and Bridger-M [22] basically just repeated the single-*k* strategy for several different *k* values, which are excluded from discussion here). As shown in Table 1, the memory occupied by these three strategies is significantly correlated with the read length and the data size. These three strategies cost the most memory on the sample that contains 0.1 million reads with length of 100bp, which indicates that the read length greatly affects the memory usage. Table 1 also shows an interesting phenomenon that the simplified suffix tree strategy occupies less memory than the single-*k* strategy and the multiple-*k* strategy on the sample that contains 1 million reads with length of 50bp. We attribute the surprising performance of the simplified suffix tree strategy on this sample to the operation of compressing the reads with the same sequence into one read. With the increase of data size, the number of reads with the same sequence increases. The read compression step significantly reduced the data size of this sample.

### Splicing graph construction

The splicing graph used by DTA-SiST is similar to that defined in Bridger [22], BinPacker [21], and IsoTree [24]. A splicing graph is a directed acyclic graph, in which the vertex represents a part of an exon or an exon. An edge exists between two vertices only if these two vertices come from a same transcript. Simple paths in such graphs usually represent plausible transcripts or parts of them.

Briefly, DTA-SiST constructs splicing graphs as follows: DTA-SiST first sets the reads whose coverage exceeds the average as seeds, and then selects an unused seed as the main contig and extends the contig with help of the suffix trees. DTA-SiST explores the suffix trees along the edges marked with the characters scanned from the endpoints of the contig successively to get the candidate read that holds the longest overlap with the current contig’s terminus. When there are multiple candidate reads, DTA-SiST selects the candidate read whose coverage is closest to that of the contig. DTA-SiST repeats the above candidates selection and extension steps until there are no candidate reads that hold at least *l*-character overlaps with the contig’s terminus. When the contig cannot be extended in either direction, DTA-SiST makes the branch extensions to construct splicing variants. DTA-SiST applies a similar strategy described in our previous work [24] to construct splicing variants and trim the splicing graph.

**Fig. 4:**
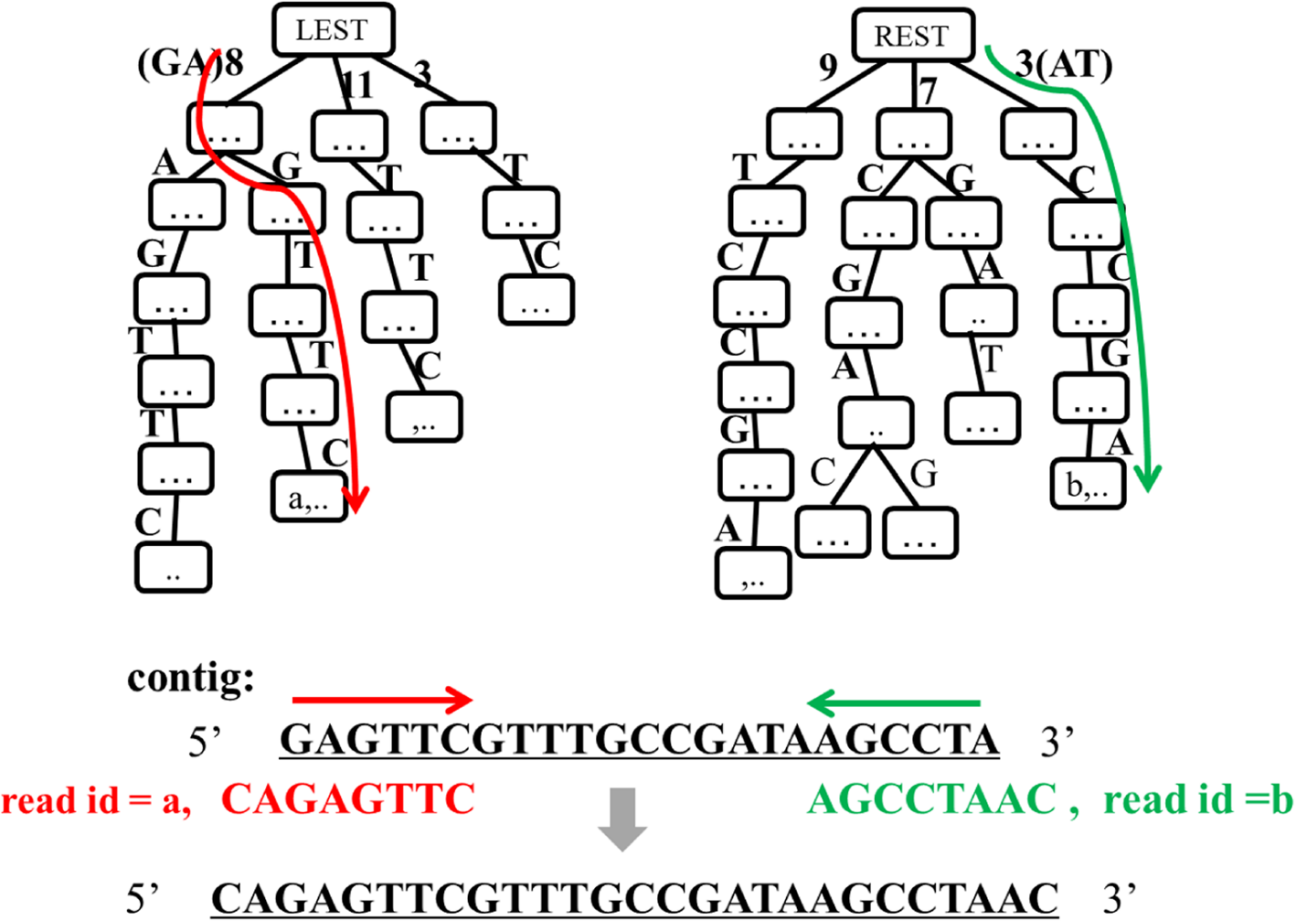
Contig extension. An example for extending contigs by reads under the help of right extension suffix tree and left extension suffix tree

Figure 4 gives an example for extending contigs by reads under the help of right extension suffix tree and left extension suffix tree. For the left extension (from 3’ to 5’): DTA-SiST searches the left extension suffix tree with characters from 5’ to 3’ successively, i.e., 8 (simplified code for GA), G, T, T, C, G, ⋯. There is a path of 8(GA)GTTC in the left extension suffix tree but no path of 8(GA)GTTCG. Hence, 8(GA)GTTC is the maximum overlap of reads with the contig’s 5’ terminus. DTA-SiST takes out the read id (*a*) stored in the last node in the path of 8(GA)GTTC and extends the contig by the read *a*. Similarly, DTA-SiST can find the candidate reads that hold the maximum overlap with the contig’s 3’ terminus through the right extension suffix tree. The contig is then extended in right direction.

The main difference between DTA-SiST and IsoTree for contig extension is the method of finding the candidate reads that hold the longest overlaps with the contig’s terminus. IsoTree extracts the candidates by trying the overlap lengths from *L*−1 to *l*. To get the reads that hold *x*-character overlaps with contig’s terminus, IsoTree first sets the first and the last *k*-mers of the *x*-character terminus as tags. Then, IsoTree searches the hash table to find the reads that contain these two tags in *O*(*a*_*x*_+*b*_*x*_) time, where *a*_*x*_ and *b*_*x*_ are the numbers of reads that contain the two tags, respectively. If an obtained read exactly has a *x*-character overlap with the contig’ terminus, it is identified as a candidate read. Once a candidate read is obtained, no smaller overlap length will be further tried. Thus, if the maximum overlap length is *x*, IsoTree will cost $O(N\sum \limits _{i = L-1}^{x}{(a_{i}+b_{i})i})$ time to find the candidate reads. Notice that DTA-SiST can find the candidate that has the longest overlap with the contig’s terminus in *O*(*L*) time for each extension and thus *O*(*NL*) in total. The suffix tree strategy is more time-saving considering that the time for building the suffix trees is *O*(*N*(*L*+*l*−1)(*L*−*l*)), which is usually far smaller than $O(N\sum \limits _{i = L-1}^{x}{(a_{i}+b_{i})i})$. Besides, although DTA-SiST needs more memory to hold the suffix trees, its memory consumption is endurable as described in “Results” section.

### Transcripts assembly

In the transcript-representing path detection step, most of researchers formulated it as an optimization problem with objective of either extracting the minimum number of paths to cover the whole graph or ensuring the minimum gaps between the coverage of the edges entering and leaving the nodes. Wondering whether the real world follows these optimization principles, we developed two new strategies for transcript-representing path extraction.

In order to detect as many transcripts as possible, DTA-SiST develops a depth-first enumeration strategy to recover all the possible transcripts that can be represented by the splicing graph. Besides, DTA-SiST develops a strict criterion to exclude the fake transcripts (these rules are generally adopted in most transcript-assembly methods): (i) the transcript sequence should be longer than 200bp (by default); (ii) the coverage of each transcript must be larger than 2 (by default); (iii) at least 20 reads (by default) can be mapped to the transcript; (iv) the whole transcript sequence must be covered by paired-end reads when paired-end reads are available.

Additionally, considering that thicker paths (paths with higher coverage) are more likely to be transcripts, DTA-SiST proposes a hybrid strategy to extract the longest path among the thickest paths. The hybrid strategy iteratively extracts paths with the compromise of length and thickness by a dynamic programming method until all the edges are covered by these paths or a predefined condition is met. The hybrid strategy based on length and coverage is also a good juxtapose to evaluate the depth-first enumeration strategy.

In the following discussion, let *G*(*V,E*) denote the splicing graph. DTA-SiST adds a source vertex *s* and a sink vertex *t* into the graph, and connects *s* (or *t*) with the vertices without incoming edges (or outgoing edges). DTA-SiST applies the function *w*(*node*) (or *w*(*edge*)) to weigh the vertex sequences (or the edge sequences) by the number of reads per base. Specifically, DTA-SiST assigns the weight of edge (*s,v*) as the weight of node *v*, assigns the weight of edge (*u,t*) as the weight of node *u*, assigns the weight of node *s* as the sum of the weights of edges leaving node *s*, and assigns the weight of node *t* as the sum of the weights of edges entering node *t*.

**Depth-first enumeration strategy.** DTA-SiST enumerates the paths from the source vertex *s* to the sink vertex *t* by a depth-first search strategy (Algorithm 1). The algorithm starts from the source vertex *s*, and iteratively traverses an unexplored edge leading from a vertex already reached. Once it encounters the sink vertex *t*, the algorithm will output the path. The algorithm uses a stack to store the nodes on the current depth-first search path, so that the path can be extracted by scanning the stack. The algorithm has a time bound of *O*((*V*+*E*)*p*), where *p* is the number of paths starting from *s*. 
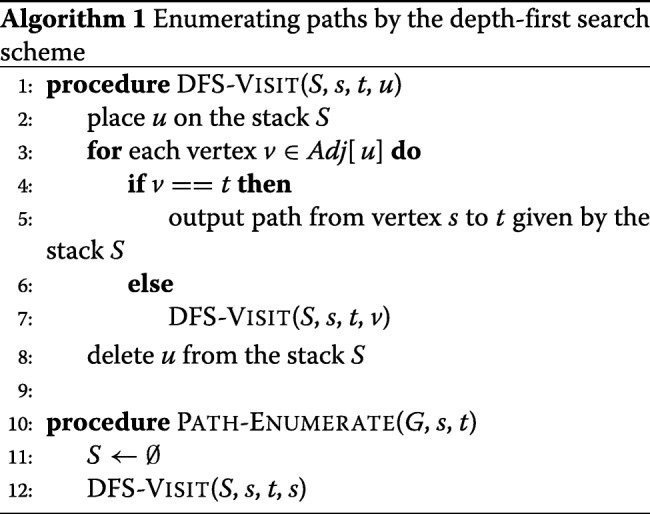


**The hybrid strategy based on length and coverage.** DTA-SiST iteratively calls Algorithm 2 to find the longest path from the paths that are extracted with the maximum coverage strategy until the path meets a pre-given empirical condition. Once a path is derived, it will be deleted from the splicing graph, i.e., the coverage of all the edges and vertices along the path will be reduced by the minimum coverage of the edges and vertices along the path. The time consumed by this strategy is *O*((*V*+*E*)*E*). 
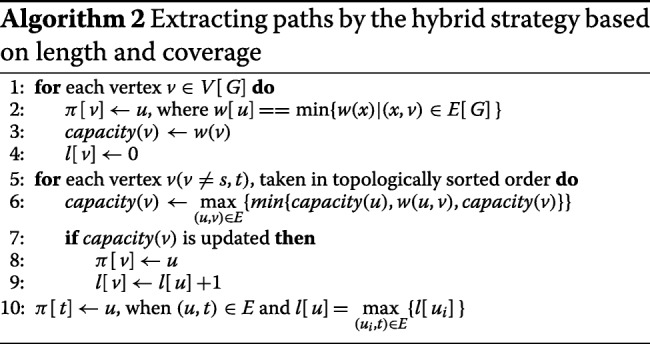


As shown in Algorithm 2, it introduces *π*[*v*] to denote the predecessor of vertex *v*. By using backtracking, a path with the compromise of length and thickness can be extracted in one iteration according to *π*[*v*]. For each node *v* in topological order, *capacity*(*v*) denotes the maximum coverage among all the paths that start from vertex *s* and end at vertex *v*, where the coverage of a path is defined as the minimum weight among all the vertices and edges in the path. Algorithm 2 computes the *capacity*(*v*) for each vertex *v* in topological order in a dynamic way until it has computed *capacity*(*t*). Besides, the variable *l*[*v*] denotes the length of the path from vertex *s* to vertex *v*.

## Results

We realized DTA-SiST in two versions: DTA-SiST-E and DTA-SiST-H corresponding to the depth-first enumeration strategy and the hybrid strategy based on length and coverage, respectively. We compared them with six state-of-the-art de novo assemblers including IsoTree (version 1.0), Trinity (version 2.3.2), BinPacker (version 1.0), SOAPdenovo-Trans (version 1.03), IDBA-Tran (version 1.1.1), and Oases (version 0.2.8) on both simulated and real datasets. We carried out the experiments on a server with 256GB of RAM and E5-2620V3*2 CPU processor.

### Datasets

In order to explore the sensitivity of assemblers on the length of reads, we used FluxSimulator [28] to simulate 11 samples with read lengths of 50*bp*,60*bp*,70*bp*,80*bp*,90*bp*,100*bp*,110*bp*,120*bp*,130*bp*,140*bp*, and 150*bp*, respectively. The only difference between these simulated samples is the length of reads. Each sample contains 0.1 million paired-end reads that are generated from 100 isoform transcripts originated from 41 different genes in chromosome 1 (CRCh38.83, NCBI).

We retrieved a dog dataset and a human dataset from NCBI SRA database, with Accession Code SRX295047 and SRR3692633, respectively. In the dog dataset, there are totally 30968059 paired-end reads with length of 50*bp*. The human dataset contains total of 43675886 paired-end reads with length of 75*bp*. The reads both in the dog dataset and the human dataset are single strand-specific. Besides, we obtained total of 62516 and 46993 annotated transcripts from UCSC for dogs and humans, respectively.

### Evaluation criteria

In this paper, we aligned transcripts predicted by assemblers to annotated transcripts by blast+ [29]. We define the full-length reconstructed transcript as an assembled transcript that holds at least 95% sequence identity to an annotated transcript. The full-length identified transcript represents an annotated transcript with at least 95% sequence covered by an assembled transcript.

On the simulated datasets, we applied recall and precision to measure the performances of the de novo assemblers. The recall is defined as the ratio between the number of full-length identified transcripts and the number of annotated transcripts, while the precision is defined as the ratio between the number of full-length reconstructed transcripts and the number of assembled transcripts.

As the annotated transcripts of the real datasets are usually not the ground truth expressed transcripts, the recall and precision defined above are not suitable to the real datasets [10, 24]. For the real datasets, we use the number of full-length identified transcripts to represent the recall, and we measure the precision by comparing the number of full-length reconstructed transcripts and the number of assembled transcripts (i.e., candidate transcripts) [21, 22].

### Simulated data

**Fig. 5:**
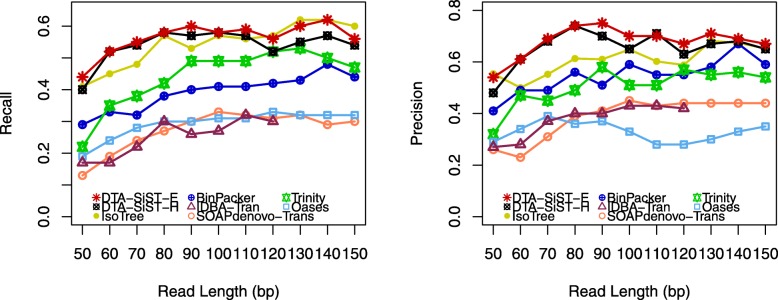
Impact of the length of read on the performances of assemblers

On the simulated datasets, besides the recall and precision, we also evaluated the sensitivity on read length of our method and the other leading approaches. We implemented all assemblers on the simulated datasets whose read lengths fall in the scope of 50*bp*∼150*bp* (Fig. 5). We ran all the compared assemblers with the *k*-mer length of 25*bp* and other parameters as default.

Figure 5 shows that DTA-SiST-E performed more competitive than the other compared de novo assemblers especially with precision measure. The average precision obtained by DTA-SiST-E on these 11 simulated samples was 0.68, which had 36.0, 25.9, 11.5, 74.4, 106.1, 78.9, and 4.6% increase over that achieved by Trinity (0.50), BinPacker (0.54), IsoTree (0.61), SOAPdenovo-Trans (0.39), Oases (0.33), IDBA-Tran (0.38), and DTA-SiST-H (0.65), respectively. DTA-SiST-E’s outstanding performance with precision benefits from the transcript filtering criterion. For the recall measure, DTA-SiST-E performed better than the other de novo assemblers except that IsoTree led a little with read lengths of 120*bp*,130*bp*, and 150*bp*. We attribute the outstanding performances of DTA-SiST and IsoTree to their read-based contig extension strategies. They extend contigs by reads while most other de novo assemblers extend contigs by *k*-mers.

From Fig. 5, we observed that the performances of the depth-first enumeration strategy DTA-SiST-E are better than those of the hybrid strategy DTA-SiST-H with both recall and precision measures, which is interesting since there is usually a tradeoff between these two measures.

### Real data

On the real datasets, we evaluated the de novo assemblers by counting the number of full-length identified transcripts and the number of full-length reconstructed transcripts. In terms of resource requirements, we assessed the de novo assemblers with regard to the running times and memory usages.

The numbers of full-length identified transcripts, full-length reconstructed transcripts, and candidate transcripts collected by the de novo assemblers are shown in Table 2.

**Table 2:** Number of full-length transcripts recovered by the de novo assemblers

Assembler	Dog dataset			Human dataset		
	Identified	Reconstructed	Candidates	Identified	Reconstructed	Candidates
Trinity	1017	1663	96018	1913	3039	437730
BinPacker	1149	2601	73419	1491	3449	192674
IDBA-Tran	598	1011	69757	1376	2196	182651
SOAPdenovo-Trans	1005	1006	85028	/	/	/
Oases	530	957	113361	1762	3126	439865
IsoTree	1354	2974	81597	2015	3821	218269
DTA-SiST-E	1504	3916	103461	2175	4930	278255
DTA-SiST-H	1370	2514	71356	1950	3959	199642

From Table 2, we observed that DTA-SiST always performed more competitive than the other de novo assemblers on recovering the full-length identified transcripts (estimate of recall). On the dog dataset, DTA-SiST-E recovered total of 1504 full-length identified transcripts and improved IsoTree (1354), Trinity (1017), BinPacker (1149), SOAPdenovo-Trans (1005), IDBA-Tran (598), and Oases (530) with 11.1, 47.9, 30.9, 49.7, 151.5, and 183.8%, respectively. Except DTA-SiST-E, DTA-SiST-H and IsoTree outperformed all the other de novo assemblers in terms of recovering full-length identified transcripts. The outstanding performances of DTA-SiST and IsoTree with recall measurement benefit from their read-based extension strategies. They extend the contig by reads while the other de novo assemblers extend the contig by *k*-mers. The *k*-mer-based extension strategies can only guarantee *k*−1-length overlaps while the read-based extension strategies ensure at least *l*-length overlaps. Both the theoretical conclusion and the experimental results show that the read-based extension strategies perform better than the *k*-mer-based extension strategies.

On the dog dataset, DTA-SiST-E, DTA-SiST-H, IsoTree, Trinity, BinPacker, SOAPdenovo-Trans, IDBA-Tran, and Oases obtained total of 3916, 2514, 2974, 1663, 2601, 1006, 1011, and 957 full-length reconstructed transcripts out of 103461, 71356, 81597, 96018, 73419, 85028, 69757, and 113361 candidates, respectively, which suggests that DTA-SiST-E also outperformed its competitors with precision.

On the human dataset, DTA-SiST maintained its superior performance on recovering the full-length identified transcripts. DTA-SiST-E obtained the most number of full-length identified transcripts (2175), followed by IsoTree (2015), DTA-SiST-H (1950), Trinity (1913), Oases (1762), BinPacker (1491), and IDBA-Tran (1376). However, DTA-SiST-E guessed more candidates than IsoTree and DTA-SiST-H. We attribute the large number of candidates collected by DTA-SiST-E to its enumeration strategy. Although DTA-SiST-H, IsoTree, and Trinity recovered almost the same number of full-length identified transcripts (1950, 2015, and 1913, respectively), DTA-SiST-H guessed the least number of candidate transcripts (199642 vs 218269 and 437730, respectively). Besides, Trinity collected more full-length identified transcripts than BinPacker on the human dataset while BinPacker detected a larger number of full-length identified transcripts than Trinity on the dog dataset.

Additionally, DTA-SiST-E, DTA-SiST-H, IsoTree, Trinity, BinPacker, IDBA-Tran, and Oases collected total of 4930, 3959, 3821, 3039, 3449, 2196, and 3126 full-length reconstructed transcripts out of 278255, 199642, 218269, 437730, 192674, 182651, and 439865 candidates on the human dataset. From these numbers we can figure out that DTA-SiST-E not only holds a superior performance with recall measurement, its precision is also higher than the other assemblers except DTA-SiST-H. We conclude that DTA-SiST-H is applicable to the high precision demand while DTA-SiST-E is suitable to the general demand.

Throughout the experiments on both simulated and real datasets, we found that most assemblers’ performances decreased as the magnitude of the data increased. The magnitudes of the simulated samples are far less than those of the real datasets, and all the assemblers performed better on the simulated datasets than on the real datasets. In addition, the human dataset is larger than the dog dataset, and almost all the assemblers performed better on the dog dataset than on the human dataset. The outstanding performance of DTA-SiST-H with precision becomes obvious when the data size increased. When the data size is big and the users focus more on precision, DTA-SiST-H is the best choice. Otherwise, DTA-SiST-E is applicable to most cases.

**Fig. 6:**
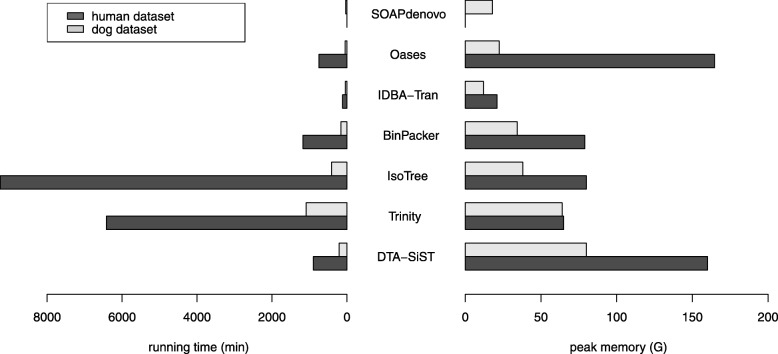
The running time and peak memory for each de novo assembler on the real dataset

We examined the assemblers’ computational demands of running time and peak memory. From Fig. 6, we can see that DTA-SiST (notice that since each splicing graph has only very limited number of *s*−*t* paths, the difference between the time cost by DTA-SiST-E and DTA-SiST-H can be ignored) cost much less time (about half on the dog dataset and 0.11 on the human dataset) than IsoTree while DTA-SiST performed more competitive than IsoTree in most cases. Although DTA-SiST (the difference between the memory cost by DTA-SiST-E and that by DTA-SiST-H can be ignored) consumed more memory than IsoTree, its memory consumption is endurable on both dog dataset (peak memory: 80G) and human dataset (peak memory: 160G which is even smaller than that 164.7G of the *k*-mer-based strategy Oases).

## Discussion

Experimental results showed that DTA-SiST performs more competitive than the other compared de novo assemblers especially with precision measure. We owe this improvement to the read-based contig extension strategy and the transcript extraction methods. However, there are still quite a few issues need to be studied further. First, despite the outstanding performance of read-based contig extension strategy on precision and speed, it needs more memory to keep the suffix trees. More elegant implementations of the suffix trees or new data structures for assisting realizing read-based extension strategy are expected. Second, due to sequencing error and the giant gap in isoform expression level of the same gene, the number of erroneous reads originating from high-expressed transcripts may be larger than that of the correct reads originating from low-expressed transcripts [30]. These erroneous reads may introduce artificial branches in splicing graphs and thus introduce chimeric transcripts while low-expressed transcripts may have little support from reads and thus are missed from the splicing graphs. High quality sequencing data and more exquisite algorithms are needed. Third, some transcripts that originate from different genes may share a long subsequence, which results in a large mixed graph (a mixture of splicing graphs that represent different genes). Once the common subsequence is longer than a read, it is hard to split the mixed graph into splicing graphs for different gene loci. In this case, a mixture graph will be seen as a splicing graph, and it will lead to a higher possibility to obtain pseudo transcripts and a lower opportunity to extract all truth expressed transcripts. To overcome this problem we need longer high quality reads and fine tuned transcript extraction rules. In short, there are still a lot of works need to do on de novo transcriptome assembly.

## Conclusion

De novo transcriptome assembly is a challenging problem that arises with the development of RNA-seq. In this article, we proposed a new approach for contig extension. We applied suffix trees of reads to quickly find the candidate reads that have the longest overlaps with contigs’ terminuses, and extended the contigs by these reads directly. We also developed two strategies to extract the transcript-representing paths in the splicing graphs: a depth-first enumeration strategy and a hybrid strategy based on length and coverage. We ran and compared these two strategies with other leading de novo transcriptome assemblers on both simulated and real datasets. The experimental results provide a whole picture of the superior performance of DTA-SiST with the cost of acceptable more memory. Future work includes developing smaller-sized data structure while keeping the searching speed and transcript extracting algorithms with higher recall and precision.

## Data Availability

DTA-SiST with choice for either strategy is available at https://github.com/Jane110111107/DTA-SiST. The real datasets were downloaded from https://www.ncbi.nlm.nih.gov. The annotation transcripts were downloaded from https://genome.ucsc.edu.

## Abbreviations

LEST: Left extension suffix tree
NCBI: National Center for Biotechnology Information
REST: Right extension suffix tree
RNA-seq: RNA sequencing
SRA: Sequence read archive
UCSC: University of California SANTA CRUZ
